# Unconscious learning processes: mental integration of verbal and pictorial instructional materials

**DOI:** 10.1186/2193-1801-2-105

**Published:** 2013-03-12

**Authors:** Seffetullah Kuldas, Hairul Nizam Ismail, Shahabuddin Hashim, Zainudin Abu Bakar

**Affiliations:** School of Educational Studies, Universiti Sains Malaysia, Penang, 11800 USM, Malaysia; Department of Foundation Education and Social Science, Universiti Teknologi Malaysia, 81310 UTM, Johor Bahru, Johor Malaysia

**Keywords:** Learning processes, Conscious processes, Unconscious processes, Mental representation, Instructional material, Working memory, Emotion, Motivation

## Abstract

This review aims to provide an insight into human learning processes by examining the role of cognitive and emotional unconscious processing in mentally integrating visual and verbal instructional materials. Reviewed literature shows that conscious mental integration does not happen all the time, nor does it necessarily result in optimal learning. Students of all ages and levels of experience cannot always have conscious awareness, control, and the intention to learn or promptly and continually organize perceptual, cognitive, and emotional processes of learning. This review suggests considering the role of unconscious learning processes to enhance the understanding of how students form or activate mental associations between verbal and pictorial information. The understanding would assist in presenting students with spatially-integrated verbal and pictorial instructional materials as a way of facilitating mental integration and improving teaching and learning performance.

## Introduction

How perceptual, cognitive, and emotional processes are interconnected as a way of facilitating or inhibiting human learning is a recurring issue in educational and psychological studies. Learning processes mainly refer to the interconnections between perception, memory, language, imagery, emotion, and motivation that allow students to mentally build connections between verbal and pictorial information patterns or between new and prior memories and integrate them with relevant knowledge structures in long-term memory (Mayer and Moreno 2003). Acquired knowledge structures refer to learning outcomes (Mayer 1989). A thorough understanding of how students mentally form the connections or construct knowledge structures is important for the improvement of teaching and learning performance (Ifenthaler and Seel 2011; Weinberger and Fischer 2006). For the better understanding, further clarification of cognitive and emotional learning processes towards the integration of knowledge, skills, attitudes, and task competence is needed (Baartman and De Bruijn 2011).

Learning processes and outcomes can be conscious and unconscious. The unconscious processes range from registering information in the sensory memory to mentally forming associations within or between information patterns and activating associative memory networks, including individual expectations, beliefs, and desires (Kowalski and Westen 2005). The unconscious can conduce to the acquisition, access, and application of knowledge without deliberate and controlled attention (Ashby and Maddox 2005; Dienes and Perner 1999; Evans 2008). On the contrary, a conscious learning process starts by deliberately paying attention to instructional materials, noticing similarities and differences between words and their particular meanings with the help of relevant prior experience, thereby mentally building coherent connections between them and organizing them into new knowledge structures (Boshuizen and Schmidt 1992; Schmidt 1990). Thus, either conscious or unconscious learning is primarily a combination of mental processes, referred to as a knowledge acquisition process, bringing memories into the mind, forming associations, retaining, and using them (Mayer and Moreno 2003). A permanent change in mental associations (Ormrod 2003), in long-term memory (Schnotz and Kürschner 2007; Sweller and Sweller 2006), or a potential change in human behaviour is considered to be learning (Walker 1996).

When humans consciously or unconsciously engage in learning activities inside or outside the classroom setting, the effect of their potential emotions on learning processes and outcomes is inevitable (Kowalski and Westen 2005; Moreno 2010). Emotion is deemed to be a reaction to significant events or stimulus that prepares action readiness or emotional behaviours (Scherer 2009). The human brain operates automatically and rapidly to emotional stimuli, thereby generating unconscious responses (Bargh and Morsella 2008; Öhman 2002; Whalen et al. 2004). Emotions are largely elicited by unconscious evaluations (appraisals) of subjective emotional experiences (Arnold 1960; Scherer 2001). Appraisal processes are characteristically intuitive, immediate, and mostly outside conscious awareness (Arnold 1960), and are not typically the product of conscious reasoning processes (Robinson and Clore 2001). For instance, the appraisal process underlying emotional attention, does not necessarily require a complex cognitive calculus, but often occurs automatically, unconsciously, and effortlessly (Scherer 2009). Unconscious appraisals are mostly related to intrinsic properties of a stimulus, such as pleasantness or unpleasantness, and to individual needs, values, and goals (Scherer 1999). These appraisals generally lead to a motivational effect (Scherer 2009). Emotional processing “acts as the on/off switch to motivation, which is the process by which goal-directed behavior is initiated and sustained either consciously or unconsciously” (Moreno 2010p. 137). Emotions evoked during learning affect the ways students learn, such as perceiving their own ability to learn, and guiding their attitudes toward engaging in similar learning activities (Howe 1998).

Thus, emotional/motivational processes intervene in instructional influences on learning activities either consciously or unconsciously (Moreno^2010^; Moreno and Mayer 2007). This intervention leads to challenges for conducting teaching and learning activities. To overcome such challenges, an essential educational objective is to render students conscious learners, allowing them to become conscious of their behaviour and learning outcomes. However, the bulk of perceptual, cognitive, and emotional processes, and the organization of human memory are too complex to be dealt with consciously; therefore, conscious learning is unlikely to occur all the time (Lewicki et al. 1992). Conscious learning can be eased by unconscious learning processes that promptly and continually establish connections between the perceptual, cognitive, and emotional processes of learning, thereby facilitating the mental integration of verbal and pictorial instructional materials (Kuldas et al. 2012). If the verbal materials are properly loaded with relevant imagery values in the use of their contents and contexts, students can, consciously or unconsciously, form coherent mental representations, manipulating complex verbal and visual information into an easy-to-grasp format (Paivio 1991). Accurately designed instructional materials ease the construction of mental representations, which in turn facilitate knowledge acquisition (Ifenthaler and Seel 2011; Seel and Blumschein 2009). However, mental representations may unconsciously evoke task-irrelevant thoughts and unnecessarily load visual and verbal working memory capacity. Moreover, the spatial integration itself can lead to an unnecessary load (Sweller et al. 1998). To control cognitive loads, students must be provided with an optimized instructional design (Sweller 2010).

To enhance the understanding of how human learning occurs and what leads to the challenges of conscious learning, the unconscious learning processes need to be brought to light (Thompson 2004). Recognition of different forms of knowledge and associated psychological processes is essential for a proper understanding of the human mind (Disessa 2002). For a better understanding, the primary aim of this article is to examine unconscious learning processes and outcomes, particularly in relation to the mental integration of pictorial and verbal instructional materials. This examination is broken down into five sections. First, the role of unconscious processes in organizing the human memory and learning is set out. Second, the unconscious activation of associative memory networks and its effect on cognitive learning processes is scrutinised. Third, the effect of unconscious mental representations on the acquisition of verbal and pictorial information is elaborated. Fourth, the relation of unconscious mental representations to conceptual learning processes is highlighted. In the final section, conscious and unconscious processing capacity for the mental integration of instructional materials is collated.

## How human memory acquires information: Conscious and unconscious learning processes

One of the most challenging problems in educational and psychological studies on human memory and learning is related to the role of conscious and unconscious processes (Schmidt 1990). In particular, existing literature provides scanty knowledge about how students consciously intervene in unconscious memory and learning processes, including their wishes, needs, beliefs, and conflicts, thereby guiding their emotional and motivational behaviours (Gilhooley 2008). The less controversial view in this regard is that perceptual, cognitive, and emotional processes are carried out unconsciously at the outset, and afterwards, possibly reach conscious processes (Bargh and Morsella 2008). Humans cannot constantly be in control of information encoding, storage, and retrieval processes or readily access and manipulate the unconsciously processed information. On the contrary, unconscious processes are not similarly limited. Unconscious processing appears to be structurally and functionally much more sophisticated than the conscious by accessing and influencing conscious processes (Bargh and Morsella 2008).

The common view on how human memory acquires information is that a limited amount of sensory information (visual, auditory, olfactory, tactile, kinaesthetic, or gustatory) is temporarily held in short-term memory, herein organized by working memory, and, in part, permanently stored in long-term memory (Baddeley 1992). Short-term memory is transitory memory storage in which the working memory exercises a restricted capacity for performing a limited amount of information or for organizing possible connections between and within information patterns (Hoffman et al. 2008). Through this limited capacity alone, students are unlikely to encode, store, and retrieve every feature of information consciously; they are likely to process and discern multiple patterns of information unconsciously, parallel to the conscious processing (Lewicki et al. 1992).

As a matter of fact, conscious processes (i.e. voluntary and controlled attention, awareness, and intention) are not persistently active to formulate or evaluate all perceived information. Therefore, they are not essentially prerequisite in discerning every bit of information at the outset, but later are required to adjust, adapt, or integrate a limited amount of information patterns into one another (Anderson 1991; Anderson and Milson 1989; Schmidt 1990; Walsh and Anderson 2009). In addition, any consciously or unconsciously formulated or discerned pattern of information does not mean it is a complete or infallible evaluation (Jou 2011). Emotional (e.g. fear, anger, pleasure, hopes, or desire) and sensory information are not amenable to a complete evaluation. Even the conscious evaluation process itself, respecting desirable and undesirable values of information, can distract the conscious mind from working on each experience (Kim and Rehder 2011).

The distinction between cognitive processes that are unconscious with constantly active functioning capacity and those that are conscious with limited functioning capacity refers to dual processing accounts of the human mind (Evans 2008; Stanovich 1999), such as implicit and explicit (Reber 1976), automatic and controlled (Schneider and Shiffrin 1977), experiential and rational (Epstein 1994), intuitive and analytic (Hammond 2010), holistic and analytic (Nisbett et al. 2001), heuristic and analytic (Evans 2006), heuristic and systematic (Chen et al. 1999), associative and rule based (Smith and DeCoster 2000), adaptive unconscious and conscious (Wilson 2003), and impulsive and reflective (Strack and Deutsch 2004). In general, relevant evidence indicates that cognitive unconscious processes can lead to the unconscious acquisition of knowledge and freely influence conscious learning processes and outcomes, but later on, this influence can be amenable to a conscious intervention (Evans 2008). Learning processes are partially amenable to a conscious intervention, such as conscious awareness, conscious control, conscious intention, or deliberate attention (Anderson 1992; Anderson et al. 2004). A good part of information acquisition or the processes of human learning, ranging from perceptual information-processing to speech production, are not necessarily subject to conscious processing (Hasher and Zacks 1984; Jacoby et al. 1992).

Cognitive learning processes are often manipulated by perceptual (Andrade and Deeprose 2007) and emotional unconscious processing (Epstein 1994). Perceptual unconscious processing can be conceived as automatically perceiving and holding a restricted amount of sensory information patterns as well as perceptually priming existing memories. Perceptual processing can lead to the activation or retrieval of verbal memories even when one undergoes a surgical operation or general anaesthesia, that is, without his or her conscious awareness (see Andrade and Deeprose 2007; Deeprose and Andrade 2006; Deeprose et al. 2004, 2005). In these studies, retrieval of the memories was detected after perceptual priming (i.e., auditory priming), presenting a word masked in background noise during surgery to detect the extent to which the subjects could identify accurately the word after the surgical operation. Either subliminally or supraliminally perceived stimuli can activate or direct perceptual and cognitive responses when people are awake, alert, and attentive without one having conscious awareness, control, or intention of encoding, storing, and retrieving information (forming mental associations), referred to as cognitive unconscious processing (Kihlstrom 1987). The mental formation of association within or between information patterns can be unconsciously made in respect of their meanings, that is, to automatically engage in the identification and categorization of similarities among meanings as well as to insert consciously made meanings in the ones that are unconsciously made (Fu et al. 2010). During the formation or making meaning process, that is, before constructed meanings are fully reflected in the conscious mind, humans are unconsciously guided by their past experiences, emotional states, expectations, fixed habits, and preferred thoughts (Gilhooley 2008). They can unconsciously generate emotional responses to an event or word stimulus based on relevant past experiences (Smith and DeCoster 2000). The generation of affective responses to a stimulus and motivation for a related behaviour, such as approach or avoid, outside of conscious processes, refers to emotional unconscious processing (Chen and Bargh 1999). These perceptual, cognitive, and emotional unconscious processes can facilitate or inhibit each other’s performances in information processing and learning activities (Kuldas et al. 2012).

In particular, a conscious intervention in emotional processing, controlling the effect of desires, hopes, or fears on cognitive processes, on the ways one think, behave, or learn, is unlikely to happen all the time (Bargh and Chartrand 1999; Bargh and Ferguson 2000; Bruinsma 2004; Meyer and Turner 2002). In addition, mainly due to the limited capacity of visual-sensory information-processing; humans cannot consciously and simultaneously encode multiple information patterns; they must unconsciously supress or discard some visual patterns of information (Kastner and Ungerleider 2000). If the patterns are associated with negative emotions, human unconsciously exert perceptual defence against them, supressing or even blocking sensory information that is emotionally disturbing (Dahl 2001; Pratto and John 1991; Robinson et al. 2004; Taylor 1991). These perceptual unconscious processes can activate several cortical areas of the brain and bring wishes, fears, and changes into feeling states (Blanco and Soto 2009; Öhman and Soares 1998; Siegel and Weinberger 2009). These effects can mediate motivational and inferential processes that produce inaccurate judgments or decisions (Efklides 2006). However, to sustain or reflect on the outcomes of these processes (e.g. making a choice, a goal pursuit), conscious control can be required.

As a result, humans are frequently unaware of how they form and activate memories, being unconscious of forming associations between memories and their retrieval processes (McCabe et al. 2011; Sid and Stanislas 2007; Siegel and Weinberger 2009; Sohn et al. 2005; Weinberger and Westen 2008). Conscious processing has a limited capacity to acquire information instantaneously and ceaselessly, and therefore, is likely to engage in preliminary unconscious information-processing stage (Barsalou 2003; Libet 1999). Hence, the conscious processing is unlikely to result in any kind of cognitive learning by obviating the need for the cooperation of unconscious processing. This assertion is further elaborated upon in the following section.

## The effect of unconscious activation of associative memory networks on cognitive learning processes

Human memory consists of information-processing stages, such as encoding, storage, and retrieval, which are consciously or unconsciously associated with one another (Wong et al. 1997). Associated memories are mentally represented in the form of a network (Anderson 1995). Through this associative network, primed memory is likely to spread activation to closely or remotely connected memories, referred to as unconscious associative priming (Ratcliff and McKoon 1981). Therefore, a perceived information pattern can evoke pleasurable or unpleasurable thoughts (Bunce et al. 1999; Westen 2006). Humans unconsciously tend to avoid unpleasant thoughts and maintain pleasant ones (Epstein 1992, 1994). The human mind can arrange thought processes without conscious intervention (Westen 1998).

This spreading activation serves to make associative memory networks more available for further cognitive processing, facilitating access to memories (Anderson 1995). Unconscious associative networks, such as beliefs, wishes, thoughts, and unconscious procedures, such as emotions, motives and defences, guide human behaviour, affecting feeling states, flows of thoughts, and behavioural tendencies (Westen 1999). When the unconscious activation of a network leads to positive (e.g. curiosity) and negative emotional states (e.g. mild anxiety), they facilitate learning performance. However, they inhibit learning when give rise to intense negative emotional states (e.g. anxiety, panic, and insecurity) and related thoughts such as incompetence belief (Kuyper et al. 2000).

Associative processes are also termed “implicit processes” instead of unconscious processes; but, it is not very clear how implicit processes differ from the unconscious since both of them work associatively in forming memories and learning processes. Both the implicit and the unconscious processes are unintentional, and utilize the same associative network of memory and procedures to mould feelings, thoughts, and behaviours (Gilhooley 2008; Westen 1999). Therefore, the implicit and the unconscious can be used interchangeably to refer to the formation or activation of association within or between networks.

The unconscious associative network, without the effective intervention of conscious awareness, conscious control, or conscious intention, can result in implicit learning (see Bunce et al. 1999; Cleeremans et al. 1998; Eraut 2000; Esteves et al 1994; Guo et al. 2011; Lewicki et al. 1987, 1992; Reber 1989, 1992; Rieber et al. 2004). However, Hammonds (2006) argued that implicit learning occurs without verbal expression but not without attention; it demands either voluntary or involuntary attention (Hartman et al. 1989; Willingham and Goedert-Eschmann 1999). As such, further investigations are needed to understand how implicit learning processes are dissociated from explicit learning processes, thereby explaining how both of them require attention (voluntary or involuntary), occur through associative networks, and significantly influence feelings, thoughts, and behaviours. Due to the difficulty of such dissociation, the absence of verbal expression as the absence of voluntary attention is likely to be detected in a related study.

Unconscious learning allows people to successfully cope with the complexity of their learning tasks (Lewicki et al. 1992), as well as with the activities of acculturation and socialization, implicitly acquiring social skills with respect to tolerable social behaviour, attitudes, and cultural worldview (Lewicki et al. 1987; Reber 1992). Learning how to speak a native language, how to listen and interact with others, and how to use general problem solving strategies are some examples of unconscious learning, which is particularly conceived to be easily, rapidly, and unconsciously acquired biologically primary knowledge (Sweller and Sweller 2006). Primary knowledge structures are information categories that human have evolved to acquire and use for processing biologically secondary knowledge, such as learning to read and write (Geary 2002). Humans have gradually evolved to deal with secondary knowledge; hence, the manner in which listening and speaking develop differs markedly from the manner in which reading and writing is learned (Sweller and Sweller 2006). The unconscious acquisition and application of primary knowledge are formed evolutionarily prior to conscious learning activities, and are not necessarily subject to conscious control or conscious intention when they promptly and continually produce responses to surrounding stimuli, rely heavily on recognising an information pattern, improve the complexity of information, or identify and disregard random elements (Seger 1994). The outcomes of these activities are more durable in memory, less affected by cognitive insults (e.g. brain injury, dementia, or amnesia) and relatively unaffected by errors or missing data (Seger 1994). However, the outcomes or the activities may not be completely be detached from voluntary or involuntary attention and working memory capacity.

Thus, cognitive learning processes are not solely restricted to the interference of conscious processing, conscious intention, conscious control, or conscious awareness (Cleeremans et al. 1998; Scott and Dienes 2010). Conscious processing can be ineffective in instantaneously and continually guiding the learning processes from the first microsecond to see, hear, taste, or to feel (Anderson 1992). The following section serves to further elucidate these arguments.

## Human learning processes in the absence of conscious interventions: The role of unconscious mental representation

Human sensory organs and the brain do not simply perceive information patterns in a dispersed manner from the very beginning, but rather in a selected manner, rendering human organisms functional before conscious intervention. The brain does not receive disorganized reports from the sensory organs; the reports on perceived information patterns are probably not scattered across the brain aimlessly or randomly (Geary 2002). Human sensory organs and brain activities can be task oriented (e.g. human survival) in the organization of reports (Geary 2002; Sweller and Sweller 2006).

Whilst humans are unconscious of how information is being organized, their organisms, such as the brain and sensory organs, seem to be conscious of their tasks, the information they form and the places to which they report on that information. Therefore, without resorting to a conscious process, such as intention, to raise questions to perceived information, one can unconsciously ask questions and seek answers without knowing the underlying factors of these behaviours (Chartrand and Bargh 1996; Greenwald and Banaji 1995; Jacoby et al. 1992). However, the recognition of both questions and answers may need a conscious process (e.g. controlled attention or conscious intention). Both conscious and unconscious processes can associatively initiate and sustain goal directed behaviour, such as decision making (Aarts and Van Den Bos 2011; Custers and Aarts 2010). This conscious interference in the associative activities does not mean that the conscious processes can completely deactivate automatic or unconscious processing in the brain to organize perceptual and cognitive processes of learning (Aizenstein et al. 2004).

It is improbable that humans immediately and persistently prioritize conscious processes to evaluate and verbalize personal experiences, either positively or negatively. There should be an automatic evaluation system for information processing when the conscious processes are not performing effectively. Relying on auto-positive evaluations, humans tend to confront what is being experienced, but avoid experiencing what was auto-negatively evaluated (Epstein 1994). These auto-evaluations can spontaneously construct and reactivate mental representations, which, in turn, build an interaction within and between patterns of subjective experiences (Andersen et al. 1995). Spontaneously constructed mental representations can guide thoughts and behaviours (Seel 2003) and, thus, be responsible for the evaluation of information in the absence of effective conscious interference (Cohen et al. 1996). The interference of mental representations is not instantaneously accessible to the conscious processes, nor is it readily transformed into verbal expressions (Disessa and Sherin 1998). Although mental representations can be constructed from both words and images; they are heavily influenced by the latter rather than the former (MacDonald et al. 1992; Trueswell et al. 1994).

One of the forms of mental representation is imagery (Paivio 1986). Imagery can be a visual mode of the brain state (Kosslyn et al. 2001). However, it is not merely a visual mode, but also a mode of quasi-visual phenomena. The quasi-perceptual experiences in other sensory modes underlie kinaesthetic imagery, haptic imagery, or olfactory imagery (see Bensafi et al. 2003; Klatzky et al. 1991; Stevens 2005). Mental imagery manifests through the perception of a real or unreal form of a physical object. Without the perception of a physical object, the imagery is unlikely to take place in the brain (see Kosslyn 2005; O'Regan and Noë 2001; Pylyshyn 2003).

Mental imagery can bring motivational and manipulative effects of memories into memory processes (Kuldas et al. 2012). Imagery formation and its effects on the information-processing stages may be monitored by unconscious and conscious intention; the latter can be based in a preliminary stage of the former (Henningsen 2010). Mental imagery can motivate people to achieve their desires, even if such a desire is not actually present to their senses (Kavanagh et al. 2005). However, such a desire should address to a physical object unlike verbal representations of abstract concepts, such as angle or God. The imagery can enable people to avoid unpleasant thoughts that unintentionally entered into whatever they remember, expect, or learn (Faw 1997; Marks 1999). Imagery affects thought processes regardless of whether they refer to past or present personal experiences (Paivio 1991). Therefore, imagery should be considered as a certain type of mental representation, and not merely as a form of past experience.

In consequence, unconscious mental representations can be operative, either in the absence of or in the presence of conscious processes, thereby reducing conscious effort in the preparation and evaluation, acceptance or rejection of information patterns, such as making meaning of concepts. Thus, the unconscious processes can organize memory, conceptual meanings, and consolidate the conscious processes of learning.

## The role of unconscious mental representations in conceptual learning processes

Human learning is manifested through multidimensional but interacting perceptual, cognitive, and emotional processes. Any kind of learning is unlikely to occur effectively without the interaction between prior knowledge and cognitive abilities, and the emotional and motivational states of learners (Moreno 2010; Seel 2001). This interaction allows humans to grasp the reality of experiences, to generate ideas and concepts, and to set personal goals or interests. It can be frequently mediated by unconscious mental representations, providing the memory with information forming at the abstract and concrete levels based on both present and past experiences (Brewer and Schommer-Aikins 2006; Marks 1999; Sadoski et al. 1997). These effects of unconscious mental representations are one of the less controversial issues in psychology as well as in philosophy (Abell and Currie 1999; Von Eckardt 1988).

Unconscious representations can facilitate the integration of illustrated verbal and visual information with one another (i.e. the integration of maps, charts, graphics, diagrams, film strips, slides, or pictures with texts in textbooks, classroom presentations, instructional manuals, and with computer based instructions), referred to as an imagery style of learning (Clark and Paivio 1991; Kuldas et al. 2012). The learning style can render learners assistance in interpreting and absorbing the meanings of information in an accelerating and retaining manner (Chun and Plass 1996; Kulhavy et al. 1993; Sadoski et al. 1997; Rieber 1990, 1991), improve their problem solving, creative, and critical thinking skills (Paivio 1986). To this end, instructional designs should properly be aligned with students’ expertise levels in cognitive learning tasks to avoid overburdening their working memory capacity (Schnotz and Kürschner 2007; Wallen et al. 2005).

Unconscious representations can play a facilitatory role in the acquisition of conceptual knowledge and thus in the occurrence of conceptual learning (Ziori and Dienes 2006), provided that educators pictorially illustrate concepts in a manner closely referring to their contextual meanings (Paivio 2007). Mental representations allow students to acquire and make meanings consciously or unconsciously (Winn 1987), requiring more or less consciously accessible knowledge about what to acquire (Dienes et al. 1991; Perruchet and Pacteau 1991). However, this requirement does not mean that making meaning of concepts with the help of their pictorial illustrations necessarily depends on effective conscious intervention (Gambrell and Bales 1986; Paivio 2007). Deliberate interventions may remain ineffective in forming a connection between dissimilar features of illustrations; whereas, it can be relatively effective in the case of similar features (Borst and Kosslyn 2008).

Illustrations help students to visualize the proper meaning of presented concepts (Dechsri et al. 1997; Heinich et al. 1999) before relying on their conceptual knowledge (Laufer and Sim 1985). Conscious knowledge cannot be instantaneously or constantly accessible and applicable, mainly due to the limited deliberate attention span and working memory capacity (Unsworth and Engle 2007). On the contrary, an unconscious association between meanings and pictures of presented concepts can be formed through unconscious associative networks because they are not necessarily dependent on working memory capacity (Anderson 1995).

If a concept does not properly refer to its common structural properties, such as form, content, context, object, and subject, there may appear many interpretations or several other potential meanings which can be made from distinct mental representations of these properties (Clark and Paivio 1991; Schnotz 1993). As students explore the illustrations of these structural properties to find relevant information under specific guidance, they will grasp comprehensive and persuasive meanings of what is being presented, resulting in effective teaching outcomes (Lowe 1996; Stringer and Irwing 1998). Presenting information that is familiar to students or relevant to their prior knowledge can allow them to recall the relevant content of information (Ranzijn 1991; Wolfe and Woodwyk 2010), even though students might infer another meaning onto which they have imposed personalized interpretations or preferences (Vermunt 1998). Accordingly, the extent to which information about a concept closely or directly refers to its form, content, context, subject, or object, may facilitate its mental representation, thereby helping students to retain and rehearse it more easily.

Rieber (1994) identified the common structural properties of concepts with attentional, affective, cognitive, and compensatory functions. The attentional function detects possible similarities between formerly and newly learned concepts (Anderson 1993). Such a recall may affect emotions and attitudes, referring to the affective function, which is discerned by the cognitive function. Students may have recourse to the compensatory function to decode the discerned affective function. The more concepts are concrete, the better the understanding of their contents can be (Beishuizen et al. 2002) and the easier the visual illustrations of the common structural and functional properties representing their meanings can be achieved (Benson 1997).

However, most probably there is no particular mental representation and meaning permanently localized for a corresponding concept in the human mind (DiSessa 2006). The representation of a concept is variable during one’s life span and might simply be affected by cognitive abilities, personal beliefs and experiences, and cultural worldviews, as well as one’s age group and gender. The representations of the relationship within and between the common structural (i.e. form, content, context, object, and subject) and functional (i.e. attentional, affective, cognitive, and compensatory) properties of concepts vary over time and from one situation to another. Accordingly, optimal meanings of concepts are not necessarily made from the ways students prefer to visualize the common structural and functional properties of concepts (Heinich et al. 1999). Instead, students can better understand concepts, which are strongly associated with their properties more than those that are less or not associated at all (Koren 1999). Such strong associations increase the likelihood of retention and the facilitation of recalling concepts (Chun and Plass 1996; Mayer 1997; Mayer and Anderson 1992). This can be because of the imagery traces of verbal definitions; concepts appropriately loaded with imagery values, which are highly associated with the common structural and functional properties (Danan 1992).

This imagery learning generally refers to the visual learning style that can enable students to integrate the common structural and functional properties with each other through representational, referential, and associative processes (Paivio 1991). Through representational processing, a stimulus can implicitly activate corresponding memories. For example, the concrete word “rose” can set the verbal memories in motion that trace and activate the corresponding image of the rose and vice versa, or an abstract word “love” can launch verbal representations that raise personal expectations, happiness or disappointment, providing the visualization of a cheerful or resentful face. This cross-activation refers to the referential processing through which the associative processes may implicitly evoke many different images and words from the one intended (Rieber 1994). However, human learning can occur more easily than without the interactions between referential and associative processes (Koren 1999). Learning these concepts through the associative processes can be easier than without these associations (Mayer 2003). Therefore, conceptual learning can become more robust if it involves implicit representational, referential, and associative processes (Mayer and Anderson 1992), because they can assist students in interpreting, making meaning, and in comprehending insights into their educational experiences (Paivio 1991). The role of these unconscious processes is also readily observable in learning native languages (Chomsky 1986; Keenan et al. 1998), in getting accustomed to social norms and forming cultural worldviews and personal beliefs (Kulkofsky et al. 2010).

However, these unconscious processes might have some inhibitory effects on conscious learning processes by evoking-task irrelevant thoughts and leading students to devote their available working memory capacity to processing these thoughts. Unconscious processing can bring not only a mark of past experiences, but also evaded or forgotten thoughts, into current emotional states, behavioural tendencies, flow of thoughts, and into learning processes (Haggerty et al. 2010; Schacter 1992; Ziori and Dienes 2006). A series of studies on the use of working memory demonstrated that negative emotions (e.g., sadness, hopelessness) brought task-irrelevant thoughts into conscious cognitive activities, thereby unnecessarily loading the available capacity, diverting attention from the task, and hampering memory performance (Ellis et al. 1995, [Bibr CR58][Bibr CR59]). Similar evidence related to motivational factors of academic achievements indicated that the striving of students for the avoidance of undesirable consequences of learning or of academic task performance triggered disruptive thoughts, such as those of failure or of appearing incompetent, increased anxiety levels, and, thus, diverted their attention away from the demands of the task (Pekrun et al. 2009; Linnenbrink and Pintrich, 2002; Senko et al. 2011). However, such evidence can be very reliable and valid only if a detrimental effect of unconscious processing is detected or identified apart from the effect of conscious processing. Similarly, the non-detrimental effect of the unconscious on conscious learning processes needs to be carefully examined so that more evidence can be presented for the contribution of the unconscious on satisfactory learning outcomes.

The inhibitory effects of unconscious processing characterize a difficult form of learning, because any information can easily be encoded, stored, and retrieved by relevant stimuli; making it difficult to regulate how and what has been learnt (Anderson et al. 1998; Huettig and McQuenn 2011; Squire 1992; Thomson et al. 2010). These influences of unconscious learning are not instantaneously and always accessible to a prompt conscious intervention (Kihlstrom et al. 1992). In contrast, Aizenstein et al. (2004) argued that conscious learning processes might interfere in unconscious learning. However, it is not very clear how conscious learning could interfere in the kind of learning that eludes conscious awareness, particularly when one engages in emotional experiences, implicit thoughts, fantasies, and unconscious defences. Furthermore, not just thoughts, but thinking itself can be unconscious (Kihlstrom 2008), thereby causing more difficulty for the conscious interference to happen.

## The conscious and unconscious functioning capacity for the mental integration of pictorial and verbal instructional materials

One of the main purposes of education is to make students cognizant of how learning occurs and how it can be developed, consciously engaging in their learning and thinking activities, to achieve a desired change in their behaviour. However, students at all levels cannot consistently learn consciously (Smith 2003). Courses aimed to teach students how to process information and think consciously have not always produced durable and transferable achievements (see Chiesi et al. 2011; Garside 1996; Ten Dam and Volman 2004), although many studies have suggested that teaching students how to process and apply information can improve their higher order thinking skills (see Kuhn 1999; Kuhn and Dean 2005; Wegerif 2011).

The scanty knowledge of learners and educators about how human learning occurs can lead to ineffectiveness in learning and teaching tasks (Veenman and Beishuizen 2004; Zohar 2004). Even though students and educators acquire necessary knowledge and skills of what to do for optimal learning, they do not necessarily achieve this purpose; mainly due to the limited cognitive capacity for conscious processing (Yuan et al. 2006), accessing and applying the knowledge and skills (Nelson 1996; Veenman et al. 2004). Therefore, both educators and students must rely on unconscious processing not only to encode, store, and retrieve information, but also to construct and reactivate mental representations (Chen and Bargh 1999; Lewicki et al. 1992). Thus, unconscious processing can compensate for the restricted capacity of conscious processing either in the absence or in the presence of conscious awareness.

Moreover, consciously constructed knowledge structures can become automated as repeatedly being applied to related cognitive learning tasks (Sweller et al. 1998). Once coherent knowledge structures (cognitive schemata) in long-term memory have been automated, students will devote less cognitive capacity to work on them (Van Gog et al. 2005). The operation of automated schemata is not significantly impeded by the capacity limitation in working memory (Van Merriënboer and Sweller 2005). “Whereas there are severe capacity limits to the amount of information from sensory memory that working memory can process, there are no known limits to the amount of information from long-term memory that can be processed by working memory” (Sweller 2004p 13). By virtue of automatized schemata “human cognitive architecture handles complex material that appears to exceed the capacity of working memory” (Paas et al. 2003p 2). Automated schemata steer human behaviour without the need to be consciously processed, and, thus, there will be available working memory capacity for other cognitive activities (Van Merriënboer and Sweller 2005).

However, neither novice nor advanced students can always consciously or unconsciously construct coherent knowledge structures or facilitatory mental representations to learn satisfactorily, particularly when their working memory capacity is overloaded or unnecessarily loaded. Such a cognitive load leads to a split-attention effect on verbal and visual representations whereby students use their available cognitive capacity without making significant contribution to their learning tasks (Casey 2003; Kalyuga et al. 1999; Mayer et al. 2001; Mayer and Moreno 2002). The overload can happen and impede the learning processes of advanced students when they are provided with redundant textual explanations for a diagram, chart, or image (Sweller and Chandler 1994). The verbal explanations are not redundant for novice students, provided that the novices are presented with sufficient images corresponding to the verbal explanation simultaneously rather than separately (Kalyuga 2012; Mayer et al. 1999; Pollock et al. 2002). For both novice and advanced students, removing the redundant explanations and keeping just the necessary pictorial representations can be beneficial for better learning (Chandler and Sweller 1991; Diao and Sweller 2007), because it is pictorial rather than textual information that facilitates the construction of coherent mental representations (Kalyuga 2012). However, if the presented images or symbols to describe the corresponding text are multiple, dynamic, and interactive, the learning processes of novices can be impeded rather than improved (Bodemer et al. 2004). Reduction in the variety of visual representations can help to moderate the involvement of distinct mental representations (Schnotz and Bannert 2003) and avoid the extraneous effort needed to map between the visual and verbal representations, allowing more cognitive effort to focus on deeper processing (Chandler and Sweller 1991, 1992). However, reduction of the cognitive load can sometimes impair learning rather than enhance it when learning tasks do not challenge the cognitive capacity of students (Schnotz and Kürschner 2007). Learning cannot only be enhanced by the reduction of a variety of different mental representations or excessive information; it must also be adapting the degree of difficulty of presented information or learning tasks to the level of knowledge and cognitive ability of students (Schnotz and Kürschner 2007).

As a result, at the outset of information processing, unconscious mental representations can reduce the conscious effort to integrate the most part of the verbal and the visual information with each other (Lewicki et al. 1992), thereby conducing to satisfactory learning outcomes (Paivio 1991). However, in most educational studies concerning the construction of teaching and learning models, there is little or no room for the educational implication of the facilitatory role of unconscious representation. Further studies have yet to be conducted to determine the extent to which unconscious mental representations inhibit or facilitate learning processes.

Finally, the effective design and application of teaching aids require educators to better understand how to facilitate the construction of meaningful mental representations enhancing learning, mainly because learning occurs when learners actively construct the representations from provided information, and well-designed instructional materials (Ifenthaler and Seel 2011; Mayer et al. 1999; Seel and Blumschein 2009). Forms of mental representations, such as mental models (representing and communicating feelings, thoughts, ideas, and personal experiences), images, concepts, and schemata, enable individuals to process information, understanding experience and events (Mayer et al. 1999). Rich and flexible mental models improve a learner’s task performance of any domain learning activity (Seel et al. 2006). Understanding an event and solving related problems presupposes either constructing the representations of relevant features to the problem or activating appropriate schemata (Ifenthaler and Seel 2011; Seel et al. 2009). Activating one of the forms allows a learner to integrate new information into pre-existing schemata (knowledge structures stored in long-term memory). An activated schema runs automatically and regulates information processing, a function which is vital for humans as it allows new information to be processed very quickly and enables them to adapt to their environment spontaneously (Ifenthaler and Seel 2011). In activating cognitive schemata, the role of motivation, emotion, and unconscious mental representations needs to be taken into consideration (Thompson 2004). It might be motivation and emotion that largely determine a learner’s cognitive performance rather than working memory capacity; the relationship between cognitive capacity, affect, and motivation has not yet been understood sufficiently (Moreno 2010).

## Conclusion

This review has served to provide an insight into the perceptual, cognitive, and emotional aspects of human learning processes and outcomes, particularly into the unconscious processes largely originated from the emotional and motivational functions of the human psyche, so as to enhance the understanding of the role of unconscious functions in the mental integration of visual and verbal instructional materials. To the aim, a set of proposals for the unconscious processes and outcomes, largely within perceptual, cognitive, social, evolutionary, educational, and psychoanalytic psychology literature, has been examined. The general consensus in the literature is that learning processes consist of interconnected perceptual, cognitive, and emotional processing stages, in which the unconscious is likely to precede the conscious. Learning processes must have an unconscious, implicit, unintentional, intuitive, experiential, or automatic processing stage, mainly due to limited conscious processing capacity. Learning processes cannot be constantly monitored at a high level of conscious processing, particularly when students engage in a motivating emotional state. The unconscious promptly works on information patterns from the first microsecond to see, hear, taste, or to feel. Conscious processing has yet to be detected in similar performance; it may be restricted because of its nature, of the limited capacity of working memory, or of unconscious processes. Therefore, an understanding of human learning processes should not be restricted to conscious learning.

Students can mentally integrate verbal and pictorial instructional materials unconsciously. Unconscious mental representations can serve as a rapid mental integration of visual and verbal information, thereby supplying information for conscious processing. Thus, unconscious processing can compensate for the limited capacity of the conscious processes, thereby laying the foundation of human learning. Unconscious representations may consolidate conscious learning processes, if verbal and visual instructional materials are spatially integrated as a way to avoid the unnecessary use of the available visual and verbal memory capacity. This avoidance, in turn, can facilitate mental representation or mental integration and ameliorate the challenge of teaching students to organize their learning processes.

Although any psychological study concerned, even remotely, with cognitive learning processes would agree that, to some extent, the processes necessarily work unconsciously, the educational implication of unconscious processing has yet to be proposed. This review suggests further examination of the contribution of unconscious processing to teaching and learning activities, inasmuch as neither educators nor students can always consciously be aware of their teaching and learning processes. The inhibitory role of unconscious processing in the integration of verbal and pictorial instructional materials might be examined with respect to the use of cognitive capacity, revealing whether this capacity is highly loaded by task-irrelevant thoughts, which are unconsciously brought into conscious processing. Such an examination would enhance the understanding of the advantages and disadvantages of unconscious learning processes in the mental integration of visual and verbal information.

## Authors’ information

Seffetullah Kuldas is a PhD student in the School of Educational Studies at Universiti Sains Malaysia. His research interests include thinking styles, conscious and unconscious processes, emotional states, and motivational factors in relation to academic achievements. Hairul Nizam Ismail is an Associate Professor in the School of Educational Studies at Universiti Sains Malaysia, where he lectures on educational psychology, cognitive psychology, thinking and reasoning, and psychological testing. His research interests include teaching strategies, problem based learning, and cognitive aspect of teaching and learning activities. Shahabuddin Hashim is a Senior Lecture in the School of Educational Studies at Universiti Sains Malaysia, where he gives lectures on educational psychology and personality theories. His research interests include personality traits, cognitive development, academic resilience, and the relation of motivational factors to teaching and learning outcomes. Zainudin Abu Bakar is a Senior Lecture in the Faculty of Education at UniversitiTeknologi Malaysia. His research interests include teaching and learning styles, cognitive and motivational theories of learning, and mental toughness.
